# Experiencing brain cancer: what physicians should know about patients

**DOI:** 10.3332/ecancer.2015.591

**Published:** 2015-11-12

**Authors:** Claudio Lucchiari, Andrea Botturi, Laura Manzini, Marianna Masiero, Gabriella Pravettoni

**Affiliations:** 1 Department of Philosophy, Università degli studi di Milano, Via Festa del Perdono 7, Milan 20122, Italy; 2 National Neurological Institute, ‘Carlo Besta’, Milan 20133, Italy; 3 Department of Oncology and Hemato-oncology, Università degli Studi di Milano, Via Festa del Perdono 7, Milan 20122, Italy; 4 Applied Research Unit for Cognitive and Psychological Science, European Institute of Oncology, Milan 20141, Italy

**Keywords:** high grade gliomas, quality of life, depression, emotional well-being, spiritual well-being

## Abstract

During the last 20 years, numerous studies have highlighted the need to consider Quality of Life (QoL) issues in the treatment of brain cancer. However, gaps in scientific knowledge are still present as we have poor data surrounding the whole experience in patients and regarding their needs.

The present study was aimed at evaluating QoL in brain cancer patients and correlated aspects. In particular, we aimed to assess QoL, mood state, and emotional issues in order to describe the patients’ experience to find out the critical aspects involved.

**Methods:**

We obtained data from 85 patients during chemotherapy treatment at the National Neurological Institute ‘C. Besta’ of Milan, Italy. We used standardised questionnaires to assess different aspects of patients’ QoL. In particular, the functional assessment of cancer therapy-brain (FACT-Br) and the Hamilton scale were used. We also performed a semi-structured ad hoc interview in order to collect ­narrative data about patients’ experience.

**Results:**

Our data depict a difficult adjustment process to the illness, even though positive elements emerged. Indeed, patients reported a satisfying self-perceived QoL, although specific concerns are still present. Further, even if many patients report depressive symptoms, only a minority have a severe condition.

**Conclusion:**

Brain cancer may heavily affect patients’ QoL and well being. However, some element of the context may improve the ­adjustment to the disease. In particular, we found that most patients found psychosocial resources to cope with cancer and that spiritual well being also seems to play a key role. These issues deserve further studies in order to obtain significant clinical recommendations.

## Introduction

Brain cancers contribute to about 2% of the cancer mortality in men and 1.4% of the mortality in women, and within the age group 15–34 years, they are the third most common cause of death because of cancer. Gliomas are classified in four grades [1] (World Health Organisation WHO 1997):

- Grade I and II named ‘low grade glioma’ (better prognosis).
- Grade III and IV are called ‘high grade glioma’ (aggressive malignant).

The most common types of brain tumours are glioblastoma multiforme (39% of all brain tumours), a high-grade (grade IV) astrocytic tumour that is almost always debilitating and rapidly fatal (6% survive two years) and anaplastic astrocytoma (grade III).

Though high grade glioma remain fatal most of the time, new therapeutic options have arisen in the last ten years, increasing the life expectancy of patients and requiring adequate scientific considerations of QoL issues. Several medical institutions have implemented studies to assess QoL in cancer patients. In particular, brain cancer patients have been the focus of a number of studies for some years, though only a few years ago QoL in brain cancer patients was a neglected issue [2] (Efficace and Bottomley, 2003). Now the situation is positively evolving, but a systematic evaluation of QoL and the correlating psychological aspects is still needed.

In this context, we used a psycho-oncological approach to examine QoL in brain cancer. Psycho-oncology has in the last two decades greatly contributed towards improving our knowledge about the QoL construct.

Many researchers in different fields proposed definitions of QoL. In 1993, the WHO QoL group proposed that QoL could be defined as the subjective perception of one’s position in life, in the cultural living environment, related to personal aims, expectations, beliefs, and thoughts [3]. This definition implies a whole consideration of patients’ needs, expectations and feelings, further than physical symptoms and functional impairment.

However, medical staff generally take into account almost exclusively the facts and the ‘elusive’ part hides behind the facts themselves. Patients are actually a combination of body and mind; they area bunch of stories, experiences, feelings, and emotions that affec t the way they approach and live a disease. The psycho-oncology approach is trying to show the patient not only as the bearer of symptoms and signs but also as a person placed in his own world, his own style, his own mental approach.

A number of well-validated and reliable QoL instruments have been standardised, with both general measures and specific modules for various tumour sites, treatment modalities, and additional dimensions like spirituality [4] (Gill & Feinstein, 1994). However, a combination of a quantitative and qualitative approach may provide a more comprehensive picture of a patient’s QoL [5, 6] (Lucchiari, Botturi, Masiero, Pravettoni, 2015; Piil *et al* 2015).

More specifically, the QoL evaluation in brain cancer patient needs particular attention. In fact, brain cancer is a life threatening illness with particular implications. Brain cancers can alter an individual’s life in a variety of ways. It could affect both motor and cognitive abilities, modify the emotional experience, and even change the personality structure [7] (Heimans & Taphoorn, 2002). Brain tumour patients may suffer from focal neurological deficits such as motor deficit, aphasia, or visual field defects. Furthermore, brain cancer activates a difficult adjustment process, involving the entire family (sometimes even the patient’s social group) who all have to cope with the motor, cognitive, or even personality changes induced by the illness [8] (Osoba *et al* 2000).

In particular, high grade gliomas (anaplastic astrocytoma or AA and glioblastoma or GBM) have a poor prognosis (with the median survival time ranging from one to three years after initial diagnosis) in which neurological impairments interact with psychological complaints in a complex manner. In fact, patients with high grade glioma have poor therapeutic perspectives, but at the same time they often undergo aggressive therapies (surgery, chemotherapy, and radiotherapy) with the aim both to prolong the survival time and to improve QoL [9] (Stupp *et al* 2005). In this difficult context, patients need to find a balance between awareness and hope, so to cope with the whole situation. This implies the activation of psychological mechanisms able to sustain a proper reaction. Hence, the impact on QoL of patients with high grade gliomas have to be analysed in the context of an illness that cannot be cured, and that often put in question the use of aggressive therapies [10] (Gilbert *et al* 2000). Consequently, an increasing amount of information included genetic features and patients’ preferences, are needed to find the best solution in a given situation. In this framework, a patient-centred model should be adopted [11, 12] (Mazor *et al* 2012; Lucchiari, Masiero, Pravettoni, 2013). However, little research is now available in this domain and most physicians, at least in Italian hospitals are advised to use a more directive paternalistic approach in this context.

We argue that a care pathway, in which is present a competent and wide monitoring of QoL of patients, including experiential aspects should help to maintain a patient-centred approach, supporting the patient’s adjustment process, as well as a good therapeutic alliance [13, 14] (Sprangers & Aaronson, 1992; Goerling, U & Stickel, 2014).

We then proceeded with a study on QoL with reference to patients with GBM or AA during chemotherapy, evaluating QoL, spiritual well-being, emotions, and physical symptoms.

In particular, our study was aimed at evaluating different aspects of the patients’ experience:

To gather data about patient’s QoL after surgery and during chemotherapy when the patient begins a therapeutic trip with higher awareness of its own situation (diagnosis, symptoms, post-surgery consequences, and therapies’ side effects)

- To explore patients’ emotions to find out their psychological resources
- To explore patient’s cognitive representation of the illness journey (including therapies)

We used a mixed methodology, coupling a semi-structured psychological interview with different questionnaires. In particular, we used the FACT-Br and functional assessment of cancer therapy spiritual well being (FACT-SW) and the Hamilton depression dcale to evaluate mood status.

## Methods

### Procedure and subjects

To evaluate the QoL of patients we used the instruments described below among a population of consecutive patients hospitalised in the Neurological National Institute ‘C. Besta’. All the patients had high-grade gliomas. We evaluated them within three months from the surgery intervention. A second evaluation was performed three months after the first. Of the 110 patients eligible and contacted for the interview, we collected complete data for 85 patients. Motivations for not accepting the assessment were the following: nine for contextual symptoms related to the treatment in progress; six for the patient refusing a psychological interview; six for refusing a psychologist-patient interview; four for organisational problems that did not permit the completion of the procedure.

The procedure used was in accordance with the ethical standard of the institutional committee on human experimentation.

The patients’ clinical characteristics are reported in Table 1.

### Instruments

The FACT-Br is a self-administered instrument made up of two parts: the core and the brain specific module [15] (Cella, 1997).

The core FACT addresses physical, family, social, emotional and functional well-being. The core instrument includes 27 items scored in a five steps Likert scale (0–4). The scores are standardised so that the maximum QoL score is 100 and the minimum 0. Each sub-scale has its own standardised score (0–100), so 4 sub-scales scores may be computed more a general one made by the sum of the 4 sub-scores.

The FACT brain module is an instrument which measures different QoL items than the core instrument [16] (Weitzner *et al* 1995). Each of the 19 items of the brain module concern a particular aspect of brain cancer, such as sight problems or being able to drive a car. The range of the scores is 19–95: a higher score corresponds to better QoL.

We also used the FACT-SW, a 12 items scale measuring the spiritual well-being of the person. This scale is not available as a standardised score, and the range of the values is 12–48.

To evaluate the depression state we used the Hamilton depression scale submitted during the interview with the patient. The Hamilton scale has 26 items concerning physical and psychological aspects particular to the depression domain [17] (Hamilton, 1960). The higher the score, the more severe the depression.

The cognitive status of the patients was assessed by the use of the Mini mental state examination corrected for age and study level (Italian version). A corrected score higher or equal to 24 is considered to be within the range of normality.

Finally, we used a semi-structured ad hoc psychological interview concerning four main domains: the emotional reactions, the familial context, the spiritual dimension, and the illness concerns. With this interview, we aimed to analyse the patients’ experience in a qualitative way so to obtain free narratives of specific life domains. The semi-structured interview is based on previous studies [18, 19] in which we collected data about the main patients’ needs and life domains by the use of standardised instruments.

### Procedure

Each eligible patient was first contacted by the clinician in charge with the aim to briefly explain the assessment procedure and to introduce the psychologist as a member of the medical staff. Patients were provided with a written explanation of the procedure and the consent to take part to the study. Once they had signed the written consent each patient was then interviewed individually by the same PhD level psychologist during the same day. The aim of this qualitative interview was to understand what mattered to patients, and their needs and expectations so as to understand their psychological and cognitive situation.

At the end of the interview, self-report scales were explained and delivered. The KPS was assessed by the physician in charge on the same day of the interview.

Patients were contacted again three months later for a follow-up evaluation. At this second session the FACT-Br and the FACT-SW scales were administered.

### Statistical analyses

The statistical analysis consisted of two steps. In the first step, we performed descriptive analyses about the subjects’ characteristics and qualitative aspects collected through the interviews. In the second step, we analysed quantitative measures (Hamilton scale, FACT scales, KPS) in order to establish associations (Pearson correlation and linear regression) between measures. Paired T tests were used to compare the first and the second evaluation (after three months) and independent T tests were used to compare quantitative variables among groups. SPSS statistical software version 20.0 was used to perform all the statistical analyses and the results were considered statistically significant when the p-value was ک 0.05.

### Qualitative analysis

A specialised psychologist conducted the interviews. Each interview was recorded and later transcribed. The transcripts were analysed and coded independently by two researchers (one involved in interviews and one not involved). The coding of the interviews were carried out following the model of content analysis for conceptual categories and emerging themes within the macro-areas already identified when interview structure was shaped. Each researcher individually analysed interviews line by line looking for emerging themes and their specific codes. Once the individual analysis was concluded, coders worked together in order to verify differences in coding and get to a convergent classification. At this stage, other researchers were also involved in order to widen the discussion and reach a broader analysis. The final classification was established and the two researchers coded each interview. This final classification was then discussed by the two researchers in order to reach a consensus. The coefficient of inter-reliability was quite high (α = 0.85), demonstrating a broadly shared vision.

We obtained a series of domain labels indicating the main themes raised during the interview. In this way for each patient we had a set of data about their experience.

## Results

Regarding the data obtained from the interview, we report here the main themes and their distributions. Four thematic illness-related domains were considered: emotional, familial, spiritual, and symptoms.

Emotional domain. The main emotions reported are: anger (25%), hope (25%); fear (30%), sadness (15%), quietness (10%). Although negative emotions are clearly present, these are generally not associated with a sense of hopelessness, since most patients report having a good self-perceived control of the situation. In this sense, the content analyses highlighted the raising of three main themes in the postsurgery period related to the ability to balance negative emotions and positive perspectives. These themes are: 1) a good physical reaction to the surgical intervention (violating previous expectations); 2) a good relationship with hospital personnel, characterized by trust and a subjective feeling of being considered as an individual with specific needs; 3) a positive perceived familial support. Most patients (about 80%) related their self-perceived ability to bring the situation under control to one or more of these themes.

Familial domain: 65% of patients reported improved relationships during the illness versus 15% who reported worse relationships. The other 20% did not recognise a significant change in the quality of the familial context (including patients with baseline poor familial relationships).

Men reported particular satisfaction in this domain. They often report being positively surprised by the familial reaction (61%) and the support received, thus finding unexpected resources.

Spiritual domain: An 80% of the patients reported themselves to be religious and to trust in God (all participants reported themselves as Roman Catholics or more generally Christians). These patients generally stated an increase in faith after the diagnosis (65%). They said they found hope and strength in their faith and generally reported more satisfaction in the practice of prayer.

Only a few patients said they had lost their faith (5%). Faith is described as an important aspect facilitating coping with illness.

Patients that did not have a religious faith reported that they believed in medicine and the development of technology and considered these to be a source of hope for their future life. We have to underline that this kind of faith is generally present in our patients (both faithless and faithful), but where a religious faith is not a present the ‘materialistic faith’ is reported as a main anchor to life. The theme of needing to have faith in something which dominates in this area.

Symptoms domain: Only a few patients did not report any symptoms or physical problems (5%). The others reported some important problem areas: seizure control (25%); therapy side effects such as nausea or vomiting (35%); motor impairments (20%); headache (10%); chronic fatigue (35%); mental confusion (15%); psycho-physical slowing (15%); sensorial impairments (15%); other symptoms (10%).

More generally, we found that about 20% of patients felt that they suffered severe impairments. However, most (55%) said they were satisfied with the overall situation (compared with their cancer illness and relative therapies). The others (25%) reported themselves to be worried about symptoms but not particularly impaired.

Finally, it is interesting to note that most participants (76%) found aggressive therapies (such as chemotherapy) less impairing than they believe to be before beginning. The subjective representation of treatments changed for the better: after the actual experience, most patients changed their mind, considering them a good weapon to fight cancer (81%).

### Quantitative data analysis

In the following table (see Table 2), we report the general and partial mean scores of the FACT instrument within three months after brain surgery (first evaluation).

The Hamilton scale showed that most patients have some symptoms related to mood disturbances and about 22% of patients suffer from light to severe depression. The depression score showed negative correlations with KPS (r = −0.383, p = 0.007), emotional well-being (r = −0.485, p < 0.001), functional well-being (r = −0.399, p < 0.001), brain concerns module score (r = −421, p < 0.001), spiritual well-being (p = 0.301, p = 0.012) and FACT-Br score (p = 0.371, p < 0.001). Depression was not correlated to age (p = 170, p = 0. 127). To verify the effect of tumour side on depression, we performed an independent T test that showed no significant differences (T (83) = 1.489, p = 0.102).

The spiritual well-being scale (FACT-SW) highly correlated with the FACT emotional well-being sub-scale (r = 0.568, p < 0.001). Furthermore, we tested the ability of psychological and physical parameters to predict the FACT score (first evaluation) by the use of a linear regression model. We used a multivariate regression model with FACT score (sum of the four main well-being subscales) as a dependent variable and brain module score (specific concerns), depression (Hamilton scale score), spiritual well-being score, age, and KPS as regressors. The result was that the spiritual well-being score is the best predictor of the general FACT score (see Table 3).

In order to evaluate if QoL parameters used for the above analyses were stable on time, at least in a short run, we performed a followup, three months after the first evaluation, using the FACT-Br. A paired T test (T (81) = 1.101, p = 0.321) did not show any significant difference, showing the preservation of QoL. The data is particularly meaningful, since only three patients dropped out (two due to organisational problems, 1 for a bad health condition). In particular, the brain sub-scale showed a negative trend while the physical, emotional, and functional sub-scale showed a positive one.

## Discussion

Our study was aimed at evaluating the subjective experience and QoL of patients with high-grade gliomas during aggressive therapies. Our data showed very interesting trends and effects, partially confirming not only the data present in previous studies but also providing original suggestions. In particular, the mean score on the total FACT scale is comparable but lower with data from other studies [5, 19, 20].

Beyond classical QoL aspects, we collected data about many aspects of patients’ experience. In particular, we were interested in subjective evaluations of specific aspects. We were then interested in patients’ psychological experience.

Qualitative data allowed us to describe our participants as well treated regarding their physical symptoms. They reported themselves to be satisfied with the overall situation and this may be the result of the positive perceived gap between poor expectations (at the diagnosis point) and the actual situation [21]. Patients seemed to be able to build a positive psychological context in which to cope with brain cancer and the related treatments. In particular, aggressive therapies were generally well tolerated as showed by the changed image of them, shifting from negative to positive. This shifting is an important target in a patient-centred medical setting, positively affecting QoL independently of the treatments’ concrete impact on survival.

An important target of our study was spiritual well being. We found it to be positively correlated with a good mood state and with the whole QoL (FACT-G score), while being negatively correlated with depression. With spiritual well being, we mean the ability of a person to find a deeper meaning in their life beyond having some religious faith. Indeed, a life threatening illness such as brain cancer put stress on spiritual and faith issues, which means that good spiritual well being in this context is a main factor in the adjustment process. This data is coherent with previous studies among other populations [22, 23, 24, 25]. Further, a high score on this scale seems to draw on the availability of psychological resources to cope with cancer. Hence, the systematic use of some assessment of spiritual well being in the QoL evaluation procedure should be taken into consideration. Even if it is difficult to figure out what specific intervention would be optimal in this domain, spiritual concerns should be addressed at least in the context of psychological support.

We also found that 22% of patients had a pathological score regarding depression in concurrence with other studies (e.g. [6]). Depression scores positively correlated with the functional status measured by the KPS. In previous studies [5] we obtained similar data but with using a self-report scale (the hospital anxiety scale). Since depression prevalence in patients with gliomas varies in studies (from 5–90%) also as a function of the instrument used (self-report versus interviews) we decided to use an interview-based instrument, in order to verify if the reporting of depression symptoms may change with the choice of diagnostic instrument [26]. Our data do not support this hypothesis, since in the two samples the prevalence rate was comparable.

Examining the relationship between depression symptoms and well-being scores, we found that mood disturbance is particularly associated with both emotional and physical aspects, showing that mood is clearly influenced by any factor that diminishes personal resources.

There are several concerns about depression evaluation in the brain cancer population [27]. At least five concerns should be taken into consideration:

- the quantity and quality of information the patient has about his/her diagnosis and prognosis
- the site of the brain lesion
- the pharmacological therapy the patient is following, in particular steroids
- the age of the patient at diagnosis
- Interaction between the factors above and the wider contextual situation (such as social support)

In our sample, during the interview most patients were able to report correctly their own diagnosis when requested. However, most of them seem to consider this diagnosis severe but not lethal (at least during the interview), and showed great trust in the physician’s work and in the development of science and technology. This means that correct information was given by clinicians, but at the same time patients interpreted it so as to cope better with their diagnosis. Considering factor b, we did not find any relationships between the lesion side and depression scores, contrasting previous findings [28]. Concerning steroid therapy, we do not have data, since all the patients interviewed were under steroid therapy. However, it is possible that steroid therapy helps the subjects to sustain their emotional well being [29]. Finally, only 5% of our patients used antidepressants at the time of the interview (SSRI).

Older patients generally show higher depression scores, pointing out the need to consider age as a possible risk factor in evaluating patients’ adjustment process. However, we did not find any significant correlation between age and depression.

Even if we considered a relatively small sample and only two near evaluation points, we found interesting suggestions about what physicians should know about patients’ experience. Our data are relative to a specific population, in which a gross total resection was possible without substantially impairing the patient’s functional autonomy, self-esteem, and coping resources. Thus, the generalisability of our data is limited to patients with similar characteristics. However, this complies with our objectives, since we aimed to study the psychological adjustment and the related QoL of patients with poor diagnosis but still able to have their own independent life after surgery and during treatment.

In these patients, we found the possibility to guarantee a self-perceived good QoL even in the presence of a life-threatening aggressive illness. We have addressed a variety of factors interacting in giving form to the self-perceived QoL: family support, physical symptoms, functional status, psychological variables, therapies, patient-physician relationship.

All these factors contribute to give rise to a protective context, where a patient may cope with the illness and also experience a good self-perceived QoL, in particular from the emotional point of view [30]. Although not all relevant parameters are directly joined to clinical activities (such as social support and psychological reactions), it is likewise important to assess them. In fact a bad adjustment may contribute to a worse response to the therapies [31]. Further, it is often possible to improve a difficult situation or a bad reaction through a variety of interventions, such as providing psychosocial support. However, to achieve the aim of helping patients to achieve their protective context, it is vital to know their subjective weaknesses as well as their strengths. We argue that only a qualitative analysis may help in this direction. Indeed, most trials addressing QoL in patients with high-grade gliomas provides relevant information about physical concerns and the whole QoL in case of aggressive therapies. In contrast, our aim was to provide health personnel with relevant information concerning critical life domains. Even if semi-structured interviews are not easy to use in everyday clinical activity on large numbers of patients, we argue that some relevant domains (in particular, spiritual, and emotional) should be addressed immediately in case of adjustment difficulties. Furthermore, our data suggest that even when patients do not report depression symptoms they may have other weaknesses, e.g. in the spiritual domain. Finally, our findings suggest that most patients are scared by aggressive therapies, but they change their mind shortly after beginning. This data may be useful for clinicians who need to talk to patients about therapies and the physical and psychological reactions they might expect.

## Conclusion

In conclusion, we think that physicians must know the whole experience of patients and not only the symptoms and signs in order to achieve the goal of delivering the best therapy available. Expectations, spiritual concerns, and emotional resources should be systematically, though briefly assessed, since standardised QoL questionnaires although indispensable are not detailed enough to really understand patients’ experience. Furthermore, physicians should learn how to use the patients’ characteristics, such as spiritual concerns, in order to improve the clinical outcome. Future research is needed to test a validated approach to this and other similar concerns in everyday activity. It also seems clear that in the case of high-grade gliomas, patients and physicians should collaborate in a patient-centred model, in which patients take their needs, preferences, and experiences to be an active part of the decision making process as already tested in other pathological conditions [32].

## Figures and Tables

**Table 1.** Patient characteristics.

	Characteriscs	No. of Patients	%
**Age, years**			
Mean	48.10	–	–
Range	27–67	–	–
**Sex**			
Male	–	46	54
Female	–	39	46
**Histology**			
GBM	–	59	69
AA	–	26	31
**Tumoure side**			
Right	–	46	–
Left	–	39	–
**KPS (Karnofsky performance scale)**			
<70	–	19	22
>=70	–	66	78
**Current treatment**			
Chemotherapy	–	85	100
Radiation therapy	25	(concomitant)	29

**Table 2.** FACT well-being sub-scales scores.

	Patients	
	Mean (SD)	Range
Physical	68.2 (12.5)	39–100
Social/familial	54.4 (8.2)	36–77
Emotional	61.5 (9.4)	41–89
Functional	51.9 (10.7)	18–76

Spiritual	30.5 (7.2)	12–48
Brain	33.5 (4.9)	20–48

FACT-G	55.3 (6.2)	41–92
FACT-Br	90.0 (8.3)	64–117

**Table 3.** Linear regression model for FACT general (FACT-G) score.

	Beta	T|	p
BRAIN	0.25	2.13	0.04
HAMILTON	0.02	0.11	0.91
SPIRITUAL	**0.35**	**2.72**	**0.01**
KPS	0.17	2.11	0.23
AGE	–0.12	2.11	0.37
